# Application Fever: Reviewing the Causes, Costs, and Cures for Residency Application Inflation

**DOI:** 10.7759/cureus.13804

**Published:** 2021-03-10

**Authors:** J. Bryan Carmody, Ilana S Rosman, John C Carlson

**Affiliations:** 1 Pediatrics, Eastern Virginia Medical School, Norfolk, USA; 2 Dermatology, Washington University School of Medicine, St. Louis, USA; 3 Pediatrics, Tulane University School of Medicine, New Orleans, USA

**Keywords:** nrmp match, residency application, eras

## Abstract

Over the past decade, the number of residency applications submitted per applicant has nearly doubled. This epidemic of “Application Fever” is expensive for applicants, burdensome for programs, and ultimately does not improve overall Match outcomes. In this review, we discuss the phenomenon of Application Fever, with a focus on contributing factors and costs of this behavior. Application Fever has its origins in the early 1990s. At that time, the number of residency applicants began to outpace the number of available positions. Because an applicant who applies to more residency programs has a greater probability of securing a residency position than an otherwise equivalent applicant who applies to fewer, "overapplication" became a dominant strategy and residency applicants began to apply to more residency programs each year. This trend was enhanced and enabled by the introduction of the Electronic Residency Application Service (ERAS). Although Application Fever is a rational decision for applicants, it imposes a substantial evaluative burden on program directors and necessitates the use of convenience screening metrics. We then briefly review potential solutions, including informational strategies, application limits, and marketplace incentives to reduce application numbers. Although a fixed cap on applications would reduce application numbers and facilitate a holistic selection process, greater transparency from residency programs regarding their selection criteria would be required to help applicants choose where to apply. To improve the residency application process for programs and applicants alike, we call upon the medical community to further study Application Fever and carefully consider solutions, including fixed application caps.

## Introduction and background

**Figure 1:**
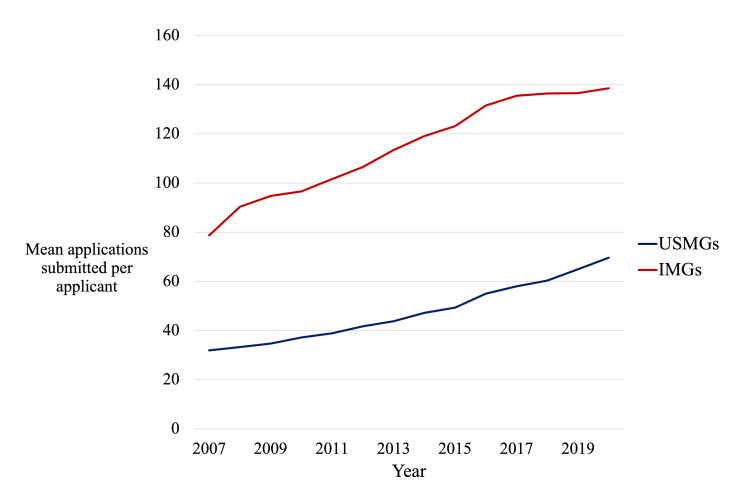
Mean number of applications submitted per applicant, Electronic Residency Application Service, 2007-2020. IMGs - international medical graduates; USMGs - United States medical graduates. Data adapted from reference [1].

For residency program directors, among the familiar rites of fall is the opening of the Electronic Residency Application Service (ERAS) - and with it, the beginning of a new residency recruitment season. But while the turning of the leaves each year is constant, each application season now brings an increasing number of applications for program directors to review. In 2020, the average U.S. medical graduate applied to 70 residency programs, while the average international medical graduate (IMG) applied to 139 [1]. Both groups of applicants apply to approximately twice as many residency programs as they did a decade ago (Figure 1) [1]. What is behind this epidemic of “Application Fever”? What does it cost us? And most importantly, what should we do about it?

## Review

Causes

The origin of Application Fever lies in the confluence of two changes to the residency application system that occurred nearly thirty years ago. In 1992, the Association of American Medical Colleges (AAMC) proposed the use of a computerized residency application system. Without a centralized system, the residency application process was cumbersome. Most programs required unique application forms, which applicants had to individually request. Given the labor involved, it is perhaps unsurprising that students applied to a median of only 12 programs [2].

Coincidentally, 1992 was the last year in which there were more residency positions available than active applicants in the National Residency Matching Program (NRMP). By 1996 - the first year ERAS was used - the ratio of positions to applicants had fallen from >1 to 0.83 [3]. At the same time that ERAS offered the means to apply broadly, a relative reduction in available positions provided the motivation to do so.

However, these factors alone are insufficient to explain Application Fever. Since the introduction of ERAS, the disparity between the number of positions and applicants has been stable - ranging between 0.75 and 0.88 - and the overall odds of matching remain favorable. In the most recent application cycle, the Match rate for U.S. seniors was ~93% (without a significant change in decades), while Match rates for international medical graduates and osteopathic seniors were at or near all-time highs [3].

Rather than basic forces of supply and demand, Application Fever is better explained by behavioral psychology and game theory. Even if the number of applicants and residency positions were perfectly aligned, not all positions are equally attractive. A candidate can gain a relative advantage over an otherwise-identical applicant by applying to more programs. When modeled mathematically, the situation approximates a prisoner's dilemma, in which overapplying becomes a dominant strategy [4,5]. Importantly, only individual candidates benefit from overapplying; overall Match rates do not improve with widespread overapplication [5].

Costs

**Figure 2:**
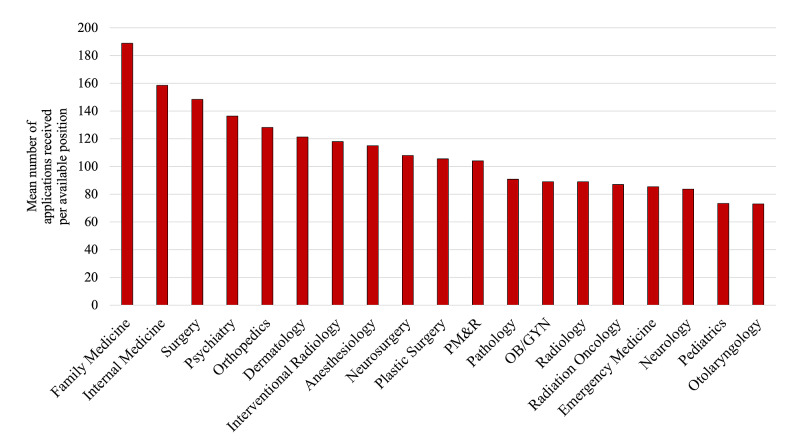
Mean number of applications received per available position, by specialty. OB/GYN - obstetrics and gynecology; PM&R - physical medicine and rehabilitation. Data adapted from reference [6].

For programs, overapplication creates a substantial evaluative burden (Figure 2). A program director who spends 10 minutes reading each application would need 10 weeks of protected time to review 1000 applications [4]. This precludes holistic review and encourages the use of readily-available metrics to screen applications.

Because United States Medical Licensing Examination (USMLE) scores are the only sortable scaled metric available in ERAS, most programs use specific target scores to limit applications for review [6]. Accordingly, medical students focus on test preparation at the expense of engagement in educational experiences that are more difficult to quantify [7-9]. Although the USMLE Step 1 exam will be reported as pass/fail as soon as 2022, 81% of program directors plan to increase emphasis on USMLE Step 2 Clinical Knowledge (CK) scores in selecting applicants [10]. This may incentivize students to focus on test preparation during clerkships at the expense of experiential learning, which could decrease readiness to provide clinical care.

Reliance on similar screening metrics (such as USMLE scores) results in programs repeatedly inviting the same narrow pool of applicants to interview. This effect can be dramatic: in both internal medicine and general surgery, 12% of applicants hold 50% of available interview positions [11]. Computer modeling demonstrates that overapplication can lead to a paradoxical increase in both unfilled programs and unmatched candidates, as programs are less able to discern sincere interest and offer interviews to the same applicants [12,13]. Overapplication may lead some program directors to decline to interview top-performing candidates, a counterintuitive decision based on the perceived low likelihood that the applicant will rank the program highly.

Application Fever is also costly. A student applying to 70 programs - the current mean for U.S. students - pays $1499 in ERAS fees alone [14]. The rising financial burden of applying to residency raises questions of equity. The expense of applying to medical school is known to deter applicants from lower socioeconomic backgrounds (who are disproportionately people from marginalized and disadvantaged backgrounds) [15]. Although application fees represent only a fraction of medical school tuition, even a slight difference in ability to pay (or willingness to incur debt) may impact an applicant's ability to match. It is potentially noteworthy that the specialties that receive the greatest number of applications per applicant - such as dermatology, ophthalmology, and neurosurgery - also have among the lowest mean educational debt and the greatest percentage of entering residents who have no educational debt at all [16]. 

Of course, application fees represent just a fraction of the total costs applicants incur. Attending in-person interviews can exceed $10,000 for competitive specialties [17-20]. As applicants have applied to more programs, they have also attended more interviews: the median number of interviews completed by a successful applicant has increased from 10 to 13 over the past decade [21,22]. These interviews are expensive: one program estimated the cost of lost faculty clinical productivity at $3,736 per interviewee [19]. Many programs may not have the capacity to interview more applicants. Yet given the decreased opportunity and financial costs of virtual interviews for students, it seems likely that applicants will choose to interview more broadly if programs provide the opportunity to do so, which will further increase costs for programs [23].

Cures

Broadly speaking, there are three types of remedies for Application Fever: informational strategies, application limits, and marketplace incentives. 

Informational Strategies

Theoretically, if applicants felt confident they would Match into their preferred specialty with a smaller number of applications, overapplication strategies may abate. The AAMC’s specialty-specific Apply Smart data purport to show the “point of diminishing returns” in residency applications [24]. However, bias in these data results in an artificially low estimate of an applicant’s likelihood of successfully entering a residency program which may paradoxically encourage well-qualified candidates to overapply [25].

The AAMC’s Residency Explorer allows applicants to compare themselves to previously-matched applicants at particular programs [26]. For example, candidates may learn that their USMLE scores place them in the lower quartile of successful applicants for a particular program, or that their research experience puts them in the upper quartile. Yet how individual programs weigh these variables is not clear. As programs move from sortable metrics toward holistic review, the inherent ambiguity of these unidirectional metrics will become even more of a limitation.

Programs should be encouraged (if not required) to be transparent about their selection criteria. For instance, programs that do not consider IMGs who require work visas, or that require certain USMLE score minimums for applicant consideration, should explicitly say so to deter applications that will be screened out. Such requirements for transparency could be enforced by the National Resident Matching Program (by making it a component of the Match Participation Agreement) or by the Accreditation Council for Graduate Medical Education (as an accreditation standard). However, unless the information provided leads applicants to a near-certain conclusion (i.e., that submitting a given application is futile), informational strategies alone will be insufficient to address Application Fever given the payoffs in the prisoner’s dilemma in which applicants are trapped.

Marketplace Incentives

If excessive applications were more costly, applicants might choose to apply to fewer programs. However, ERAS already uses a tiered fee structure, with candidates submitting >30 applications paying a small premium to do so [14]. As demonstrated by Figure 1, this has done little to impact overall application numbers. A steeper tax might discourage overapplication - but would disproportionately impact applicants with fewer financial resources, and might still not significantly alter current application strategies (given the high cost of going unmatched).

Another form of tax is to increase the time required to complete each application. The existence of a common application form facilitates a scattershot application strategy. A program-specific additional requirement - such as a brief essay - may lead to a 25% drop in applications received [27]. However, broad adoption of secondary applications may simply fuel another application arms race for students. Completing a large number of secondary applications is time-consuming, but students who complete more would enjoy a relative advantage in the high-stakes residency selection competition. This may lead to undesirable consequences such as further disengagement from educational or extracurricular activities during application season.

Other policies could attempt to reduce the value of overapplication. For instance, a limit might be placed on the number of interviews an applicant could accept [12,25]. If applying to additional programs cannot result in more interviews, well-qualified applicants might choose to apply more selectively. Interview caps would address a particularly frustrating aspect of the current system for programs: late or last-minute interview cancellations from candidates who initially accept more offers than they can attend. This approach would also curtail “interview hoarding” by top-performing applicants [11]. However, capping interviews could provide a perverse incentive to some applicants to apply to more programs to completely fill their allotment. Additionally, unless programs commit to a common interview offer date, interview caps will slow scheduling: students will be reluctant to accept or decline interview offers until they hear from all programs. Finally, like application caps, limiting interviews may be subject to legal challenges.

Another possibility might be to provide an incentive for applicants to apply to fewer programs. The Early Result Acceptance Program (ERAP) [28] and similar proposals [29,30] call for a two-round system with limited applications in the initial round. Applicants who do not match (or do not apply) in the first round may apply to unlimited programs in a second round. Because participation in the initial round is voluntary for applicants and programs, ERAP sidesteps potential legal challenges associated with fixed caps. However, applicants who do not match in the first round may feel pressure to apply widely in the second, which may increase expense and reliance on screening metrics [31]. Additionally, a two-round system may be prone to gaming. For instance, should a program rank a less competitive but highly interested applicant in the first round, or hope that they are still available in the second?

ERAP would address a byproduct of overapplication: it is increasingly difficult for program directors to identify candidates with sincere interest. However, formal preference signaling could be implemented without limiting application numbers or adding rounds to the system, as otolaryngology programs chose to do for the 2020-2021 application cycle [32]. Allowing applicants to designate a limited number of “preferred” programs at the time of application might allow programs to better gauge interest and more efficiently allocate interviews [33,34]. Although computer analysis suggests that such an approach would work [13], mathematical modeling does not account for human behavior. Preference signaling would be most valuable when applying to a “reach” program where the applicant might otherwise not have received an interview, incentivizing students to prioritize program competitiveness over genuine interest. 

Preference signaling could also paradoxically encourage overapplication by systematically de-valuing all non-preferred applications. Thus, instead of allowing applicants to designate a fixed number of preferred programs, one potential solution would be to provide each applicant 100 “points” that could be assigned at the applicant’s discretion to signal weighted interest among the programs to which they apply. Program directors could use the points assigned as a sortable metric in ERAS. Because students who overapply would have to assign each additional application a smaller number of points, excess applications become incrementally less valuable.

Application Limits

Imposing a limit on the number of applications a student can submit is the only certain way of eliminating Application Fever. Aside from occasional mention in the literature [35-37], there has been little movement to study application caps. Why?

Some do not believe application caps are fair. Caps are not imposed on college or medical school applications, for instance, and some applicants may have compelling reasons to apply broadly (such as couples matching) [35]. Others fear that such limits would lead to a legal challenge based on restraint of trade. It is beyond our expertise to comment upon the potential merits of such a suit, but involvement in litigation would be time-consuming and costly, regardless of the outcome.

To be effective, application caps must be coupled with enhanced information about programs. Applicants would no longer be able to apply indiscriminately, and would instead need to select programs carefully to maximize their individual probability of matching. Because the overall Match rate is a function of the number of applicants and the number of positions available, application caps should not change the proportion of applicants who ultimately enter residency training programs. However, there is potential for a temporary increase in unmatched applicants and/or unfilled positions given that applicants would face uncertainty in optimal application strategies under a capped system. This is likely to benefit some applicants and disadvantage others; it is difficult to predict to which group a particular applicant belongs. Careful study is required to determine whether capping applications would systematically disadvantage particular groups, and whether the systemic benefits outweigh costs to individuals. 

## Conclusions

Given the behavioral and economic incentives that underlie residency application inflation, Application Fever will not resolve on its own, and current strategies that simply encourage students to apply less are unlikely to provide relief. We believe that a fixed cap on applications is the only solution capable of cooling Application Fever. However, for application caps to be successful, they must be coupled with better information to help students choose where to apply. We support formally studying caps and other innovative solutions, and call upon graduate medical education broadly to work on solutions for this issue.
